# Choice of Surgical Procedure for Cervical Ossification of the Posterior Longitudinal Ligament

**DOI:** 10.3390/jcm11185396

**Published:** 2022-09-14

**Authors:** Toshitaka Yoshii, Kenichiro Sakai, Masaaki Machino, Takeo Furuya

**Affiliations:** 1 Department of Orthopedic Surgery, Tokyo Medical and Dental University, 1-5-45 Yushima, Bunkyo Ward, Tokyo 113-8519, Japan; 2 Department of Orthopedic Surgery, Saiseikai Kawaguchi General Hospital, 5-11-5 Nishikawaguchi, Kawaguchishi 332-8558, Japan; 3 Department of Orthopedic Surgery, Nagoya University Graduate School of Medicine, 65 Tsurumaicho, Showa Ward, Nagoya 466-8550, Japan; 4 Department of Orthopedic Surgery, Chiba University Graduate School of Medicine, 1-8-1 Inohana, Chuo Ward, Chiba 260-8670, Japan

In cervical ossification of the posterior longitudinal ligament (OPLL), spinal cord compression causes neurologic symptoms. In the early stages of the disease, most patients with OPLL may not exhibit any neurologic symptoms. As OPLL develops in size, however, the spinal cord and nerve roots become compressed anteriorly, resulting in myelopathy and/or radiculopathy. Minimally symptomatic patients can be treated conservatively, whereas those with progressive neurologic symptoms typically require surgical treatment at an appropriate time.

The optimal surgical procedure for the treatment of cervical OPLL is a matter of debate. Anterior decompression and fusion (ADF) can provide direct decompression to the spinal cord and stabilize the involved spinal segments [1]. Because the location of spinal cord compression is essentially at the anterior aspect, an anterior approach is reasonable for achieving sufficient decompression [2]. However, the procedure is complex and technically demanding. Although posterior decompression techniques, such as laminoplasty (LAMP), are relatively simple procedures, an indirect decompression may be insufficient for patients with massive OPLL and/or kyphotic alignment. For such patients, posterior decompression with fusion (PDF) is increasingly performed [3].

## 1. ADF versus LAMP

Previous studies have compared clinical outcomes for patients after ADF and LAMP; however, most studies were retrospective with a small sample size. Thus, high-quality evidence is insufficient for the optimal surgical approach in the treatment of cervical OPLL. Nevertheless, a prospective nonrandomized trial comparing the two procedures [4] showed better outcomes with ADF than with LAMP in patients with kyphosis and in patients with a high ossification occupancy ratio within the spinal canal (≥50%). Furthermore, a systematic review comparing the two procedures [5] to assess improvement in neurologic symptoms found better outcomes with ADF. ADF was superior to LAMP, particularly in patients with a high canal occupancy of OPLL (≥60%).

A meta-analysis conducted in context of the Japanese clinical guidelines [6] demonstrated similar results. Neurologic symptoms showed better improvement with ADF than with LAMP, especially in patients with a high canal occupancy (≥60%) and patients with kyphosis. Furthermore, postoperative cervical spine alignment was more favorable after ADF than LAMP. In contrast, the complication rate was significantly higher with ADF than with LAMP. The reoperation rate was also higher with ADF than with LAMP. Therefore, patients who undergo ADF can be expected to have better improvement in neurologic symptoms compared with LAMP, particularly in cases with a high canal occupancy and with kyphosis. However, the rates of complications and reoperation are higher with ADF than with LAMP.

## 2. LAMP versus PDF

The most common surgical procedure for cervical OPLL is LAMP. However, posterior decompression with instrumented fusion has been increasingly used because of recent developments in spinal instrumentation surgeries [3,7]. Although no high-level evidence exists to support the optimal procedure via the posterior approach for cervical OPLL, several retrospective studies compared LAMP and PDF.

In Japanese clinical guidelines [8], a meta-analysis was conducted to compare LAMP and PDF. Results for neurologic improvement are controversial—some studies showed significant improvement in PDF and some showed no differences between LAMP and PDF [9,10]. However, particularly in patients with K-line (–) spine and those with a high canal-occupying OPLL, the recovery rate of the JOA score tended to be better with PDF than with LAMP. With regard to the cervical alignment, the postoperative C2–7 angle was significantly more preserved with PDF than with LAMP.

## 3. ADF versus PDF

Only a few studies compared ADF and PDF for the treatment of cervical OPLL. A comparative study evaluating surgical outcomes of ADF and PDF for OPLL patients with ≥50% canal occupancy [3] showed no significant differences in the postoperative neurologic recovery rate between the two groups. Although in patients with kyphotic alignment (C2–7 angle < 0 degrees), the recovery rate was higher with ADF, approach-related complications were more frequently observed in the ADF versus PDF group. A comparative national database study also showed that ADF was associated with more postoperative complications compared with PDF, except for surgical site infection.

Overall, each procedure has both advantages and disadvantages. Based on the current data, it is difficult to simply determine which procedure is superior. Several patient factors, such as background, OPLL level, OPLL size, cervical alignment, and K-line should be considered when selecting the surgical procedure, as well as the surgeon’s skill and experience with the procedure. Furthermore, high-quality studies should be conducted to optimize the surgical procedure for each patient undergoing surgical treatment for cervical OPLL.

## References

[1] Matsunaga S., Kukita M., Hayashi K., Shinkura R., Koriyama C., Sakou T., Komiya S. (2002). Pathogenesis of myelopathy in patients with ossification of the posterior longitudinal ligament. J. Neurosurg..

[2] Yamaura I., Kurosa Y., Matuoka T., Shindo S. (1999). Anterior floating method for cervical myelopathy caused by ossification of the posterior longitudinal ligament. Clin. Orthop. Relat. Res..

[3] Yoshii T., Sakai K., Hirai T., Yamada T., Inose H., Kato T., Enomoto M., Tomizawa S., Kawabata S., Arai Y. (2016). Anterior decompression with fusion versus posterior decompression with fusion for massive cervical ossification of the posterior longitudinal ligament with a >/=50% canal occupying ratio: A multicenter retrospective study. Spine J..

[4] Sakai K., Okawa A., Takahashi M., Arai Y., Kawabata S., Enomoto M., Kato T., Hirai T., Shinomiya K. (2012). Five-year follow-up evaluation of surgical treatment for cervical myelopathy caused by ossification of the posterior longitudinal ligament: A prospective comparative study of anterior decompression and fusion with floating method versus laminoplasty. Spine.

[5] Xu J., Zhang K., Ma X., Yin Q., Wu Z., Xia H., Wang Z. (2011). Systematic review of cohort studies comparing surgical treatment for multilevel ossification of posterior longitudinal ligament: Anterior vs posterior approach. Orthopedics.

[6] Yoshii T., Egawa S., Hirai T., Kaito T., Mori K., Koda M., Chikuda H., Hasegawa T., Imagama S., Yoshida M. (2019). A systematic review and meta-analysis comparing anterior decompression with fusion and posterior laminoplasty for cervical ossification of the posterior longitudinal ligament. J. Orthop. Sci..

[7] Koda M., Mochizuki M., Konishi H., Aiba A., Kadota R., Inada T., Kamiya K., Ota M., Maki S., Takahashi K. (2016). Comparison of clinical outcomes between laminoplasty, posterior decompression with instrumented fusion, and anterior decompression with fusion for K-line (-) cervical ossification of the posterior longitudinal ligament. Eur. Spine J..

[8] Kawaguchi Y., Imagama S., Iwasaki M., Kaito T., Koda M., Chikuda H., Hasegawa T., Mori K., Yoshii T., 2019 Clinical Practice Guideline for Ossification of Spinal Ligaments Working Group (2021). Japanese Orthopaedic Association (JOA) clinical practice guidelines on the management of ossification of the spinal ligament, 2019. J. Orthop. Sci..

[9] Chen Y., Guo Y., Lu X., Chen D., Song D., Shi J., Yuan W. (2011). Surgical Strategy for Multilevel Severe Ossification of Posterior Surgical strategy for multilevel severe ossification of posterior longitudinal ligament in the cervical spine. J. Spinal Disord. Tech..

[10] Nakashima H., Imagama S., Yoshii T., Egawa S., Sakai K., Kusano K., Nakagawa Y., Hirai T., Wada K., Katsumi K. (2022). Comparison of laminoplasty and posterior fusion surgery for cervical ossification of posterior longitudinal ligament. Sci. Rep..

